# Supplementary material for: The HLA-DRB1*07 Allele Is Associated with Interstitial Lung Abnormalities (ILA) and Subpleural Location in a Mexican Mestizo Population
Source: Biomolecules. 2022 Nov 9;12(11):1662. doi: 10.3390/biom12111662 (PMC9687358; doi:10.3390/biom12111662)
Supplement: Supplementary file 1 [file biomolecules-12-01662-s001.zip › biomolecules-1999051-supplementary.pdf]

The HLA-DRB1*07 Allele Is Associated with Interstitial Lung Abnormalities (ILA) and Subpleural Location in a Mexican Mestizo Population

Ivette Buendia-Roldan, Marco Antonio Ponce-Gallegos, Daniela Lara-Beltrán, Alma D. Del Ángel-Pablo, Gloria Pérez-Rubio, Mayra Mejía, Moises Selman, Ramcés Falfán-Valencia

Supplementary material

Supplementary Table S1. Demographic and clinical characteristics of ILA subgroups.

Variables	Subpleural (n = 75)	Central (n = 15)	*p-value*
**Age (years)**	69 (60-90)	72 (58-84)	0.498
Sex, female (%)	44 (58.66%)	8 (53.33%)	0.70
**Smoking Status**			
Smoker, yes (%)	44 (58.66)	11 (73.33)	0.28
Years of smoking	20 (1-62)	40 (1-64)	0.109
Cigarettes per day	4 (0-40)	4 (0-20)	0.891
Tobacco index	2 (0-76)	10 (0-40)	0.401
**Comorbidities**			
DM	15 (20%)	3 (20%)	1
HAS	22 (29.33)	7 (46.66%)	0.18
**Pulmonary function**			
6MWT	460 (80-600)	400 (108-566)	0.066
Initial SaO2	93 (90-99)	93 (92-96)	0.596
Final SaO2	87 (54-96)	90 (67-97)	0.802
FVC %	94.50 (57-142)	81 (49-122)	0.022
FEV1	100 (68-147)	86 (54-115)	0.053
FEV1/FVC	79 (61-92)	76 (69-89)	0.664
DL_{CO}/VA	95 (57-144)	95 (77-127)	0.642
DL_{CO} adj	90 (43-116)	87 (64-111)	0.268

Supplementary Table S2. Allele frequency between groups.

HLA-DR	ILA		HC		p-value	OR	95% CI
	n = 85	F (%)	n = 220	F (%)			
DR51	9	10.59	29	13.18	0.54	0.8	0.35-1.72
DR52	26	30.59	79	35.91	0.38	0.8	0.46-1.35
DR53	48	56.47	99	45	0.07	1.6	0.96-2.62
DR54	2	2.35	13	5.91	0.19	0.4	0.08-1.74

Supplementary Table S3. Allele frequency according to ILA HRCT pattern.

HLA DRB1	GGA-ILA		RO-ILA	
	n = 20	F (%)	n = 62	F (%)
*01	2	5	10	8.06
*03	0	0	11	8.87
*04	15	37.5	31	25
*07	6	15	12	9.68
*08	4	10	19	15.32
*09	1	2.5	1	0.81
*10	0	0	2	1.61
*11	4	10	10	8.06
*12	0	0	2	1.61
*13	0	0	6	4.84
*14	1	2.5	6	4.84
*15	5	12.5	5	4.03
*16	2	5.0	9	7.26

Supplementary Table S4. Allele frequency according to GGA-ILA and HC groups.

HLA DRB1	GGA-ILA		HC	
	n = 20	F (%)	n = 189	F (%)
*01	2	5	29	7.67
*03	0	0	23	6.08
*04	15	37.5	105	27.78
*07	6	15	25	6.61
*08	4	10	62	16.40
*09	1	2.5	4	1.06
*10	0	0	4	1.06
*11	4	10	24	6.35
*12	0	0	3	0.79
*13	0	0	20	5.29
*14	1	2.5	34	8.99
*15	5	12.5	28	7.41
*16	2	5	17	4.50

Supplementary Table S5. Allele frequency according to RO-ILA and HC groups.

**HLA DRB1**	**RO-ILA**		**HC**	
	n = 62	F (%)	n = 189	F (%)
*01	10	8.06	29	7.71
*03	11	8.87	23	6.12
*04	31	25	105	27.93
*07	12	9.68	25	6.65
*08	19	15.32	62	16.49
*09	1	0.81	4	1.06
*10	2	1.61	4	1.06
*11	10	8.06	24	6.38
*12	2	1.61	3	0.8
*13	6	4.84	20	5.32
*14	6	4.84	34	9.04
*15	5	4.03	28	7.45
*16	9	7.26	17	4.52
